# Using functional near‐infrared spectroscopy to assess social information processing in poor urban Bangladeshi infants and toddlers

**DOI:** 10.1111/desc.12839

**Published:** 2019-05-17

**Authors:** Katherine L. Perdue, Sarah K.G. Jensen, Swapna Kumar, John E. Richards, Shahria Hafiz Kakon, Rashidul Haque, William A. Petri, Sarah Lloyd‐Fox, Clare Elwell, Charles A. Nelson

**Affiliations:** ^1^ Labs of Cognitive Neuroscience, Division of Developmental Medicine Boston Children’s Hospital Boston Massachusetts; ^2^ Harvard Medical School Boston Massachusetts; ^3^ University of South Carolina Columbia South Carolina; ^4^ Icddrb Dhaka Bangladesh; ^5^ University of Virginia Charlottesville Virginia; ^6^ Birkbeck College London UK; ^7^ University of Cambridge Cambridge UK; ^8^ University College London London UK; ^9^ Harvard Graduate School of Education Cambridge Massachusetts

**Keywords:** fNIRS, infant, poverty, social processing, toddler

## Abstract

Children living in low‐resource settings are at risk for failing to reach their developmental potential. While the behavioral outcomes of growing up in such settings are well‐known, the neural mechanisms underpinning poor outcomes have not been well elucidated, particularly in the context of low‐ and middle‐income countries. In this study, we measure brain metabolic responses to social and nonsocial stimuli in a cohort of 6‐ and 36‐month‐old Bangladeshi children. Study participants in both cohorts lived in an urban slum and were exposed to a broad range of adversity early in life including extreme poverty, malnutrition, recurrent infections, and low maternal education. We observed brain regions that responded selectively to social stimuli in both ages indicating that these specialized brain responses are online from an early age. We additionally show that the magnitude of the socially selective response is related to maternal education, maternal stress, and the caregiving environment. Ultimately our results suggest that a variety of psychosocial hazards have a measurable relationship with the developing social brain.

**Research Highlights**

- Children growing up in low‐resource settings and exposed to high levels of adversity are at risk for not living up to their developmental potential.
- Growing up in stressful and chaotic households may compromise the development of social information processing.
- Functional Near‐Infrared Spectroscopy (fNIRS) can non‐invasively assay different elements of social information processing.
- Here we report that the neural metabolic correlates of processing social stimuli are related to psychosocial hazards (e.g. maternal education, maternal stress, and the caregiving environment).

## 1 INTRODUCTION

Growing up in low‐resource households elevates the risk of children not reaching their developmental potential. Behavioral studies conducted with children growing up in low‐resource homes across the globe have found decreased performance on executive function (e.g. attention; behavioral inhibition) and language tasks (Codina et al., 2015; Fernald, Kariger, Hidrobo, & Gertler, 2012; Fernald, Weber, Galasso, & Ratsifandrihamanana, 2011; Hackman, Gallop, Evans, & Farah, 2015; Hamadani et al., 2014; Schady et al., 2015). Moreover, neuroimaging studies, conducted mostly in the United States, suggest that developmental, cognitive, and even long‐term educational setbacks observed among children in poor households are associated with changes in both neural structure and physiology, highlighting that these cognitive and developmental differences are neurobiologically embedded (Brito, Fifer, Myers, Elliott, & Noble, 2016; Brito, Piccolo, & Noble, 2017; Hair, Hanson, Wolfe, & Pollak, 2015; Neville et al., 2013). A key challenge when studying children growing up in low‐resource households is understanding the relative contribution attributable to the myriad of hazards children are exposed to, including undernutrition, infection, parental stress and poor parental mental health (Jensen, Berens, & Nelson, 2017).

Unfortunately there have been few studies that have examined the independent effects of co‐occurring risks. Effects of undernutrition and infection are, for instance, often intricately intertwined since chronic infection and inflammation can lead to undernutrition, and undernutrition increases children's vulnerability to chronic inflammation. Similarly, psychological stress can lead to chronic low‐grade infection, and chronic infection can affect psychological states and is a risk factor for emotional problems including depression (Jensen et al., 2017). Studies have also shown synergistic effects of maternal mood and nutrition on children's developmental, cognitive and emotional outcomes (Barker, Kirkham, Ng, & Jensen, 2013; Marques, Bjørke‐Monsen, Teixeira, & Silverman, 2015; Monk, Georgieff, & Osterholm, 2013; Pina‐Camacho, Jensen, Gaysina, & Barker, 2015). Accounting for effects of co‐occurring risks may provide insight into the independent effects of various risk exposures on children's development among children growing up in low‐resource households and can thus help guide future targeted interventions.

This study addresses the need for increased knowledge of the independent contribution of complex risk exposures to children's early cognitive outcomes by looking at processing of social compared with nonsocial and emotionally neutral information in order to explore neural mechanisms underlying the poor neurocognitive outcomes and socioemotional deficits among children growing up in impoverished homes in urban Bangladesh.

**Figure 1:**
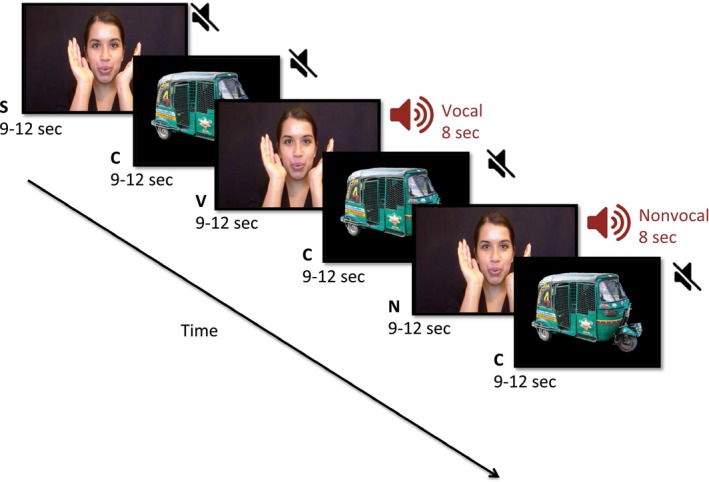
Stimulus presentation paradigm. The visual stimulus alternates between social and nonsocial stimuli. The auditory stimulus is either silence, vocal sounds (e.g. laughing, crying) or non‐vocal sounds (e.g. fan whirring, bells). The stimuli are labeled as ‘S’ for silent social, ‘C’ for control or reference nonsocial silent, ‘V’ for auditory vocal social, and ‘N’ for auditory non‐vocal/nonsocial stimuli. The visual social versus nonsocial contrast was condition ‘S’ versus condition ‘C’ The auditory social versus nonsocial contrast was condition ‘V’ versus condition ‘N’

Social and nonsocial information processing was examined via recordings of functional near‐infrared spectroscopy (fNIRS) in response to socially salient video clips and/or sounds compared with pictures of modes of transportation and natural and industrial sounds (see Figure 1). Although previous studies of children growing up in low income environments have focused on outcomes related to executive functions and language, more basic processing of social and nonsocial information can provide important insight into early developing social brain networks that begin to emerge before the age of 6 months and that continue to develop well into later childhood (Grossmann, 2015). Indeed, the experimental paradigm used in the current study has previously been used to examine the processing of auditory and visual social stimuli in both high‐ and low‐resource settings, including the UK (Lloyd‐Fox et al., 2013, 2009; Lloyd‐Fox, Blasi, Mercure, Elwell, & Johnson, 2012) and the Gambia (Lloyd‐Fox et al., 2017, 2014). We hypothesized that we would observe variability in social information processing in our cohorts of 6‐ and 36‐month‐old children. We further predicted that this variability would be partially explained by children's exposure to a wide range of poverty‐related exposures outlined in detail above. In particular, we hypothesized that the processing of social stimuli (in contrast to nonsocial stimuli) would be most strongly related to maternal psychological stress because of previous research indicating that mothers’ mental health can adversely impact the quality and quantity of social interactions the mother engages in with the child and thus directly impact the child's exposure to social emotional stimuli (Lovejoy, Graczyk, O'Hare, & Neuman, 2000). Moreover, previous studies have shown that maternal depression is associated with lower social cognitive skills (Jensen, Dumontheil, & Barker, 2014), poorer imitation (Perra, Phillips, Fyfield, Waters, & Hay, 2015) and poorer emotion recognition (Winer & Thompson, 2011) in children. Early markers of social information processing, while seldom studied in the context of poverty, are likely important for not only socioemotional outcomes later in life, but for more general cognitive functions. Indeed one study found that socioemotional development may be more impacted by poverty than cognitive development (McCoy et al., 2016).

fNIRS is portable, infant‐ and child‐friendly, requires minimal infrastructure aside from a reliable power source and relative to MRI, is inexpensive, making it particularly well‐suited to study the neural underpinnings of poverty and poverty‐related risks in low‐ and middle‐ income countries (LMICs). The method uses light in the near‐infrared frequency range to measure hemodynamic changes in the brain. Optical fibers deliver light to the outside of the head, where it diffuses through scalp, skull, and brain. Detectors placed several centimeters away on the head capture the light that has traveled through the head, and changes in light levels at the detectors indicate changes in the underlying oxyhemoglobin (oxyHb) and dexoyhemoglobin (deoxyHb) concentrations. These changes in oxyHb and deoxyHb are related to neural activity through neurovascular coupling mechanisms. The light sources and detectors are affixed to the head in a cap or band, yielding a head‐mounted brain imaging method that is relatively motion‐tolerant for small movements that are typically present in infant and child data (Behrendt, Firk, Nelson, & Perdue, 2018). fNIRS used in combination with well‐designed experimental stimuli is able to determine if participants show discrimination among different stimuli, and due to the relatively good spatial resolution of fNIRS, it can also suggest cortical regions active when infants and children process stimuli. The regions of the brain interrogated in this study are the bilateral temporal and inferior frontal regions. These regions support social cognition in general (Beauchamp, 2015; Deen, Koldewyn, Kanwisher, & Saxe, 2015), auditory processing (Abla & Okanoya, 2008), and language processing (Raizada, Richards, Meltzoff, & Kuhl, 2008).

## 2 METHODS

### 2.1 Participants

Participants lived in an urban slum in the Mirpur neighborhood of Dhaka, Bangladesh and were enrolled before or at birth for a study on diarrheal disease burden [infant cohort, CRYPTO see (Steiner et al., 2018)] or an oral vaccine efficacy trial [toddler cohort, PROVIDE, see (Kirkpatrick et al., 2015)]. Participants were recruited for the fNIRS study at the targeted ages of 6 months for infants [*N* = 130, 6.1 (*SD* 1.0) months old, male *n* = 54] and 36 months for toddlers [*N* = 130, 36.4 (*SD* 0.2) months old, male *n* = 72]. Inclusion criteria included no known neurological conditions and no known hearing or visual impairments. The study was approved by the Institutional Review Board of Boston Children's Hospital and the Ethical Review Committee of International Centre for Diarrheal Disease Research, Bangladesh (icddr,b). Parents provided informed consent before enrollment in the fNIRS study. Local staff were fully trained on data collection procedures, and data was collected at the local PROVIDE clinic building within walking distance to the participant neighborhood.

### 2.2 Stimuli

Stimuli were presented on a video screen that was located approximately 100 cm away from the child when seated on the parent's lap. Processing of social stimuli was examined in both the auditory and visual domains using a contrast of social versus nonsocial stimuli (see Figure 1), and have been used previously in high income and LMICs, and described in detail elsewhere (Lloyd‐Fox et al., 2017, 2012, 2014). Briefly, visual social stimuli were short movie clips 4–6 s long consisting of women moving their eyes, mouths, and hands as if performing a nursery rhyme. Visual nonsocial stimuli were pictures of methods of transportation, with seven pictures presented in each nonsocial block. Both social and nonsocial stimuli were adapted to be familiar to participants by showing women of South Asian descent, and modes of transportation typical in Bangladesh. Both social and nonsocial visual stimuli were presented in full color and roughly the same size on the screen. Auditory stimuli were identical to previous studies, with the social condition including communicative and non‐communicative non‐speech adult vocalizations (coughing, laughing, yawning and crying), and nonsocial condition including naturalistic environmental sounds (that were not human or animal produced, but were likely to be familiar to infants and children; running water, fans, bells). No verbal language sounds were presented. A restriction of studying auditory processing in awake infants of this age is that they need to be presented with concurrent visual stimulation in order to direct and focus their attention, which in turn is designed to reduce movement; to this end we have matched the stimulus design from the infants with the 36‐month‐old cohort. To maximize the number of valid trials, the presentation of auditory and visual stimuli were therefore combined. The experimental condition trials rotated between visual social (silent), visual social with auditory social, and visual social with auditory nonsocial stimuli. As the auditory social and nonsocial conditions were both paired with the social visual video, it is worth noting that neither auditory condition could be considered purely nonsocial stimuli. The combination of auditory and visual stimuli in the auditory trials also means that the auditory condition is inherently multimodal in contrast to the visual trials that only have one sensory modality. Experimental trials were always followed by reference trials consisting of the nonsocial silent condition. Visual stimulus blocks had lengths randomized between 9 and 12 s. Auditory stimuli were 8 s in duration with the onset at the same time as the social visual onset. Auditory social stimuli did not match the visual social stimuli, as the visual social stimuli contained gestures and mouthing words, and the auditory social stimuli was nonverbal social sounds such as laughing or crying. The parent was instructed to refrain from interacting with their child during stimulus presentation unless the child became fussy or sought their attention. Up to 10 blocks of each stimulus of interest (visual social, auditory social, and auditory nonsocial) were presented, depending on child tolerance.

### 2.3 fNIRS data collection

**Figure 2:**
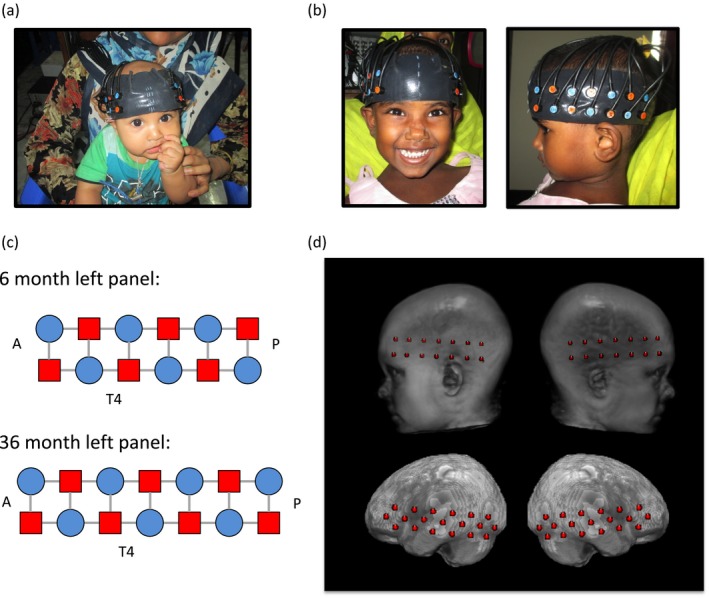
(a) Example head band placement on a 6‐month‐old infant. (b) Example head band placement on a 36‐month‐old toddler. (c) Source and detector grid. The left array is shown, the participants also had a right array of the same design. The anterior of the array is on the left, posterior on the right, sources are marked as red squares, detectors are marked as blue circles, and gray bars indicate the channel locations. (d) Upper panel shows example placement of optode locations on a 3‐year‐old head/brain atlas, Lower panel shows estimated channel locations on the atlas

fNIRS data were collected with the NTS optical topography system (Everdell et al., 2005) with wavelengths of 780 and 850 nm. Custom‐built headbands were used to position optode arrays over the bilateral temporal and inferior frontal cortices (see Figure 2), and photographs of the participants were taken after the headbands were placed to ensure proper placement and facilitate offline anatomical localization. The 6‐month‐old cohort data were collected with 12 sources and 12 detectors arranged to measure from 32 channels. The 36‐month‐old cohort data were collected with 14 sources and 14 detectors arranged to measure from 38 channels. Source‐detector separation was 2 cm and the system sampling frequency was 10 Hz. Video recordings of infant and child behavior during the experiment were simultaneously collected for offline coding of looking behavior. Participants were excluded from further analysis for fussiness (*n* = 22 6‐month‐olds, *n* = 5 36‐month‐olds), equipment failure (*n* = 2 6‐month‐olds and *n* = 1 36‐month‐old), headband that was too loose or improperly aligned (*n* = 2 6‐month‐olds, *n* = 2 36‐month‐olds), or medical condition contraindicated with study participation (*n* = 1 6‐month‐old).

### 2.4 fNIRS data analysis

#### 2.4.1 Hemodynamic response estimation

Data were analyzed using the HOMER2 (Huppert, Diamond, Franceschini, & Boas, 2009) toolbox for MATLAB (The Mathworks, Natick, MA). First, channels with low light levels (mean below 0.003 V) were automatically detected and eliminated from further analysis. Participants with more than 25% channels with low light levels were not included in further analysis (*n* = 3 6‐month‐olds and *n* = 14 36‐month‐olds). Data were converted from raw intensity values to optical density. Next, a wavelet motion correction algorithm (Molavi & Dumont, 2012) was applied with iqr = 0.5 (Behrendt et al., 2018). A bandpass filter was then applied from 0.05 Hz to 0.8 Hz, and the optical density data were converted to chromophore concentrations using the modified Beer–Lambert law with a pathlength factor of 5 (Duncan et al., 1995).

Trials were then screened for looking time before being included in a block average over condition. Participant looking behavior was coded offline using the video recordings. Only trials where the participant was looking at the screen for at least 50% of the trial were included in analysis. Participants were required to have at least three usable trials from each condition to be included in the final dataset. This criterion eliminated 11 6‐month‐old participants but no 36‐month‐old participants. The total number of participants included in the final dataset was 85 in the 6‐month‐old cohort and 105 in the 36‐month‐old cohort. A maximum of 10 trials were presented, leading to a range from three to 10 blocks included for each condition in both cohorts. The mean number of accepted trials per condition was 7.5 for 6‐month‐olds and 8.8 for 36‐month‐olds, with more trials included in the older group (*t*(174) = 4.77, *p *< 0.001). In the 6‐month cohort, participants attended to an average of 7.4 visual social trials (*SD* 2.2), 7.6 auditory social trials (*SD* 2.5), and 7.2 auditory nonsocial trials (*SD* 2.2). In the 36‐month cohort, participants attended to an average of 9.0 visual social trials (*SD* 1.7), 9.0 auditory social trials (*SD* 1.8), and 8.3 auditory nonsocial trials (*SD* 1.4).

The block‐averaged response for each condition for each participant was normalized to convert to a z‐score time‐course by dividing each timepoint by the standard deviation over the reference pre‐stimulus period. The brain response was averaged over a time window of interest to estimate a response for each condition. The time window of 12–16 s after stimulus onset was selected in line with prior studies using this stimulus (Lloyd‐Fox et al., 2017, 2018). Both oxyHb and deoxyHb responses in the time window of interest were calculated.

#### 2.4.2 Anatomical localization

**Figure 3:**
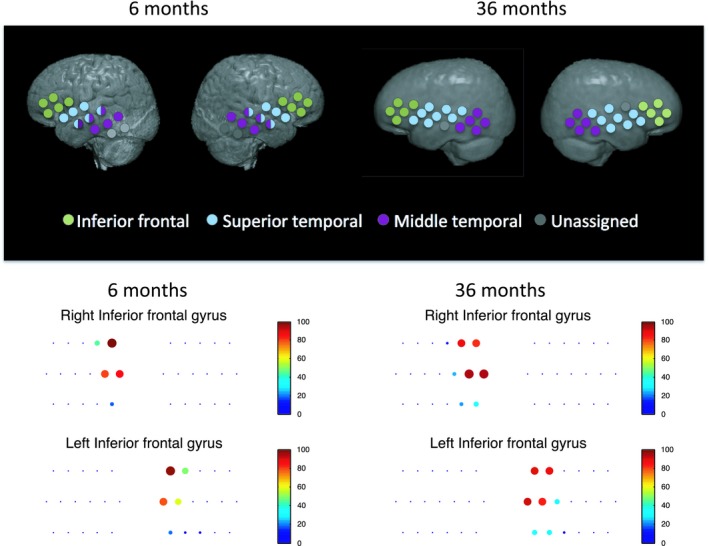
Upper panel. Approximate group‐level ROI locations shown on a group average atlas for the 6‐month and 36‐month cohorts. Inferior frontal, superior temporal, and middle temporal regions could be localized in most participants. Note that the 6‐month cohort had 3.8 channels per ROI on average and the 36‐month cohort had 4.5 channels per ROI on average. Lower panel. Channel locations for example IFG ROI along with color and size of dots indicating what percentage of participants had that channel included in the ROI

fNIRS array placement was modeled for each participant individually to estimate the brain regions of interest (ROIs) underlying the measurement channels. Participants were matched to individual MRIs in the Neurodevelopmental database (Richards, Sanchez, Phillips‐Meek, & Xie, 2010; Sanchez, Richards, & Almli, 2012) by age, head measurements (head circumference, ear‐to‐ear measurements over forehead and over the top of the head), and gender. Fiducial markers were placed on the matched MRI to correspond to participant photos, and then a previously developed algorithm was used to place a virtual array on the MRI head model relative to those fiducial markers (Lloyd‐Fox et al., 2014). Accurate placement of the optode locations on the matched MRIs was confirmed using photographs of the participant during the experiment. If accurate optode locations could not be achieved on the matched MRI, a new MRI was selected based on matching just the ear‐to‐ear measurements over the forehead of the child and the MRI. Photon propagation modeling (Fang, 2010) was used to estimate the diffuse optical tomography sensitivity functions from each source and detector pair comprising a channel, and was confirmed with previously described geometrical methods of projecting the channel location to the cortical surface, as shown in Figure 2d (Lloyd‐Fox et al., 2014; Okamoto & Dan, 2005). The intersecting cortex regions were labeled using the LONI atlas (Fillmore, Richards, Phillips‐Meek, Cryer, & Stevens, 2015). Three brain ROIs in each hemisphere could be measured in most participants—left and right inferior frontal gyrus (lIFG, rIFG), left and right superior temporal gyrus (lSTG, rSTG), and left and right middle temporal gyrus (lMTG, rMTG), and these six ROIs as defined on a participant level were used for further analyses (see Figure 3, Figure S1).

Trial‐averaged time courses were combined over channels using a weighted approach based on estimated ROI contributions for each channel. Channels that localized to more than one ROI contributed to each ROI proportionally for ROIs with more than 25% localization weights. Accordingly, channels could contribute to one, two or three ROIs with a weight of 1, ½ or 1/3 to each ROI, respectively. Furthermore, participants with fewer than two channels contributing to an ROI had that ROI excluded from analysis. In the 6‐month cohort, on average, the rMTG ROI was 5.4 (*SD* 1.2) channels, the rSTG was 3.0 (*SD* 0.9) channels, the rIFG was 3.3 (*SD* 0.8) channels, the lMTG was 4.7 (*SD* 1.1) channels, the lSTG was 3.1 (*SD* 0.8) channels, and the lIFG was 3.2 (*SD* 1.2) channels. In the 36‐month cohort, on average, the rMTG ROI was 4.8 (*SD* 1.7) channels, the rSTG was 3.9 (*SD* 1.0) channels, the rIFG was 4.5 (*SD* 1.2) channels, the lMTG was 5.2 (*SD* 1.7) channels, the lSTG was 4.0 (*SD* 1.1) channels, and the lIFG was 4.5 (*SD* 1.4) channels. The IFG localization is shown in each cohort in Figure 3. The ROI exclusion criteria led to some non‐uniformity in the number of participants included in each ROI and the resulting degrees of freedom in statistical tests.

#### 2.4.3 Risk variables included in the multivariate models

Children growing up in low‐resource settings are exposed to various hazards including undernutrition, infection, parental stress and poor parental mental health. We chose to focus on a set of seven biological and psychosocial risk factors that have been related to poor developmental outcomes. Household poverty, maternal psychological stress, and family care were assessed using home observations and standard questionnaires administered in oral interviews with mothers. Interviews were conducted by local, trained interviewers. Details about the measurement of each of the risk variables are given below.

##### Poverty

Household poverty was assessed at enrollment shortly after the child was born using items from home observations and sociodemographic questionnaires to create a cumulative score. This score included information about income‐to‐needs relative to the World Bank's definition of extreme poverty as living on less $1.9 per day, presence or absence of ‘housing risks’ related to poor construction materials, crowding, and poor sanitation and an index of family assets such as ownership of furniture and electrical appliances. Among the 6‐month‐olds, 88% of the families met the World Bank's criteria for poverty and an average family had six assets at home and was exposed to three housing risks (e.g. living with dirt floor and sharing toilet facilities). Among the 36‐months‐olds, 93% of the families met the World Bank's criteria for poverty, an average family had five assets at home and was exposed to four housing risks.

##### Maternal education

The number of years of self‐reported maternal education was used as a continuous variable. Mothers reported between 0 and 10 years of formal education. The mean length of education was 4.78 (*SD* = 3.46) years in the 6‐month‐old cohort and 4.21 (*SD* = 3.65) in the 36‐month‐old cohort.

##### Maternal psychological stress

Maternal psychological stress was assessed at the time of the neurocognitive assessment (i.e. when children were 6 or 36 months old) and was measured as a cumulative index using summary scores from three maternal questionnaires, namely a Bangla version of the Edinburgh Postnatal Depression Scale (Gausia, Fisher, Algin, & Oosthuizen, 2007), the Tension Scale, a measure of emotional distress and common mental health problems previously used with Bangladeshi women (Karasz, Patel, Kabita, & Shimu, 2013), and the Perceived Stress Scale (Cohen, Kamarck, & Mermelstein, 1983). The three measures showed good internal consistency. The Edinburgh postnatal depression scale (10 items, max. 40 points) Cronbach's α = 0.838 and sample mean [*SD*] = 7.32 [5.39] in the 6‐month‐old cohort and Cronbach's α = 0.767 and sample mean [*SD*] = 6.75 [4.34] in the 36‐month‐old cohort. The Perceived stress scale (10 items, max. 40 points) Cronbach's α = 0.674 and sample mean [*SD*] = 21.38 [6.45] in the 6‐month‐old cohort and Cronbach's α = 0.622, sample mean [*SD*] = 18.46 [5.58] in the 36‐month‐old cohort. The Tension scale (24 items, max. 96) Cronbach's α = 0.840 and sample mean [*SD*] = 40.72 [8.73] in the 6‐month‐old cohort and Cronbach's α = 0.904 and sample mean [*SD*] = 33.76 [8.96] in the 36‐month‐old cohort. The psychosocial measures were also highly interrelated in both the 6‐month‐old (Cronbach's α = 0.710) and the 36‐month‐old cohort (Cronbach's α = 0.729), suggesting that they measure a cohesive construct related to maternal stress and mental well‐being, and were summed to create the cumulative ‘maternal stress’ score.

##### Undernutrition

The child's height was assessed at the time of the study by measuring in centimetres and converted into an age referenced and standardized height‐for‐age z‐score (HAZ) using the World Health Organization's Anthro Plus software (version 3.2.2). In addition to HAZ, head circumference was also included as a risk factor that might be more specific to cognitive outcomes than HAZ (Scharf et al., 2018).

##### Inflammation

Inflammation was measured as the concentration of C‐reactive protein (CRP), a protein secreted by the liver in response to inflammation, in peripheral blood in mg/L. The 6‐month‐old infants had one CRP assessment collected at 4 months. This was log transformed to create a continuous measure. The 36‐month‐old children had five CRP assessments obtained between the ages of 6 weeks and 24 months. In line with previous publications from this cohort, we assessed the persistent inflammatory burden by summing up the number of times the child's CRP score was among the 50% highest in the sample to create a inflammation score from zero to five (Naylor et al., 2015).

##### Family care

Family care was also assessed at the time of the cognitive assessment using the Family Care Indicators (FCI). The FCI is a questionnaire that assesses stimulating activities (e.g. playing, singing, reading) that the mother, father, or ‘other caregiver’ engaged in with the child within the last 30 days. The FCI has been widely used in low and middle‐income countries including Bangladesh (Hamadani et al., 2010). The family care indicators (35 items, max. 35 points) showed good internal consistency in the 6‐month‐olds (Cronbach's α = 0.771, sample mean [*SD*] = 10.51 [4.70]) and the 36‐month‐olds (Cronbach's α = 0.891, sample mean [*SD*] = 15.02 [6.847]).

##### Statistical comparisons

Socially selective regions in the visual domain were defined as having a statistically significant difference between the silent visual condition response and the nonsocial silent reference condition, as measured using a *t* test comparing the oxyHb response to reference. Auditory socially selective regions were defined as having a statistically significant difference between the social vocal condition response and the nonsocial non‐vocal condition response, as measured using a paired *t* test comparing the oxyHb responses. While we focus on the oxyHb responses in accordance with previous studies using this paradigm and the general practice in studies with infants and young children (Lloyd‐Fox, Blasi, & Elwell, 2010), the deoxyHb visual and auditory social contrasts are also reported as some recent work has suggested that deoxyHb results may be more spatially specific (Dravida, Noah, Zhang, & Hirsch, 2018). Reported *p*‐values were false discovery rate (FDR) corrected over six ROIs for multiple comparisons (Benjamini & Yekutieli, 2001). Analyses were conducted separately for the 6‐month and 36‐month cohorts.

In an exploratory analysis, relationships between the magnitude of the oxyHb social contrasts and the seven risk variables of interest were first calculated using bivariate nonparametric correlations to screen for potential entry into an omnibus regression model. Risk variables included three measures related to undernutrition: HAZ, head circumference, and elevated inflammation, two measures related to low SES: poverty and maternal education, and two measures related to the child's social environment: maternal stress, and family care. Risk variables that were correlated with either contrast in either group at any ROI were used to construct an omnibus regression model. In the regression model, all risk factors were entered together as predictors and the dependent variable was the visual or auditory social contrast at each ROI. Robust regression was used as implemented in MATLAB. The omnibus model tested for significant relationships between risk factors simultaneously and allowed for estimation of risk factor to brain response relationships while controlling for other risk factors. The same omnibus model was applied to both age groups.

## 3 RESULTS

### 3.1 Risk factors

Descriptive statistics for the risk factors in each group are reported in Table 1. The 6‐month and 36‐month cohorts, while recruited from the same neighborhood, were statistically different in almost all risk measures as evaluated by independent samples *t* tests. The 36‐month group had lower HAZ scores [*t*(250) = 5.13, *p *< 0.001] and greater poverty sum scores [*t*(254) = 2.70, *p *= 0.007]. The 6‐month group had higher maternal stress sum scores [*t*(251) = 2.99, *p *= 0.003] and lower family care scores [*t*(253) = 13.49, *p *< 0.001]. The two groups were not significantly different in number of years of maternal education (*t*(254) = 0.79, *p *= 0.43). In the infant cohort, there was no difference in between participants who were included versus excluded in the fNIRS analysis in HAZ, poverty, head circumference, maternal education, or inflammation (*ps > *0.3). There was a significant difference in maternal stress sum scores [*t*(122) = 2.17, *p *= 0.03] with higher maternal stress scores in the included group versus the excluded group, and family care scores [*t*(123) = 2.33, *p *= 0.02], with higher family care scores in the included versus the excluded group. There were no significant differences in risk factors between participants included versus excluded in the fNIRS analysis in the toddler cohort (*ps > *0.4). There were 45 children in the toddler cohort classified as stunted based on HAZ score.

**Table 1:** Risk factor descriptives in each cohort

	6‐month‐olds				36‐month‐olds			
	Mean	*SD*	Min.	Max.	Mean	*SD*	Min.	Max.
HAZ	−1.03	0.86	−3.57	0.57	−1.65	0.92	−3.31	1.03
Poverty index	14.68	2.90	8	21	16.00	3.24	8	22
Psychosocial stress	65.71	18.50	34	108	54.76	17.69	26	105
Family care	4.41	1.82	1	9	8.07	2.85	2	12
Head circumference	42.46	1.27	38.80	45.30	47.25	1.48	43.6	51.2
CRP (log / cumulative log)	0.18	0.61	−1.00	1.31	2.53	1.29	0	5
Maternal education [years]	4.83	3.48	0	10	4.21	3.65	0	10

Abbreviations: CRP, C‐reactive protein; HAZ, height‐for‐age; Max., Maximum; Min., Minimum; SD, Standard deviation.

The relationships between pairs of risk factors in each cohort as assessed by nonparametric bivariate correlations are reported here from the subsample of participants with neuroimaging data. In the 6‐month cohort, significant correlations were seen between the poverty sum score and years of maternal education (*r *= −0.45, *p *< 0.001), and HAZ and maternal stress (*r *= 0.31, *p *= 0.011). No other significant correlations were seen between risk factors in this cohort.

In the 36‐month cohort, significant correlations were found between the poverty sum score and HAZ (*r *= −0.39, *p *< 0.001), poverty and head circumference (*r *= −0.25, *p *= 0.011), poverty and elevated inflammation (*r *= 0.28, *p *= 0.005), poverty and maternal education (*r *= −0.47, *p *< 0.001), and poverty and maternal stress (*r *= 0.20, *p *= 0.046). HAZ was additionally significantly correlated with head circumference (*r* = −0.31, *p *= 0.001), and HAZ was correlated with elevated inflammation (*r *= −0.28, *p *= 0.005). Elevated inflammation was additionally correlated with maternal education (*r *= −0.20, *p *= 0.048), and maternal education was also correlated with family care (*r *= 0.19, *p *= 0.049).

### 3.2 Neural responses

**Figure 4:**
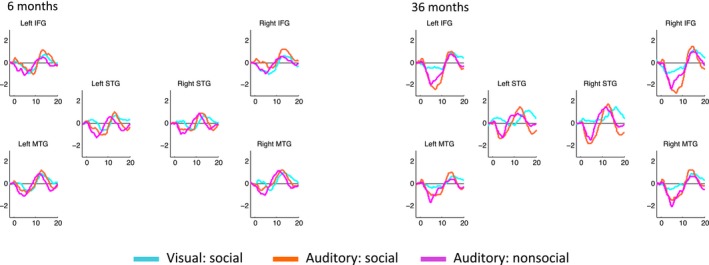
Time courses of the oxyHb response in each ROI for 6‐month and 36‐month cohorts

The time courses of the oxyHb responses for each ROI, condition, and age group are shown in Figure 4. The peak response to the stimulus is approximately 14 s in both age groups, similar to what has been found in previous studies.

**Figure 5:**
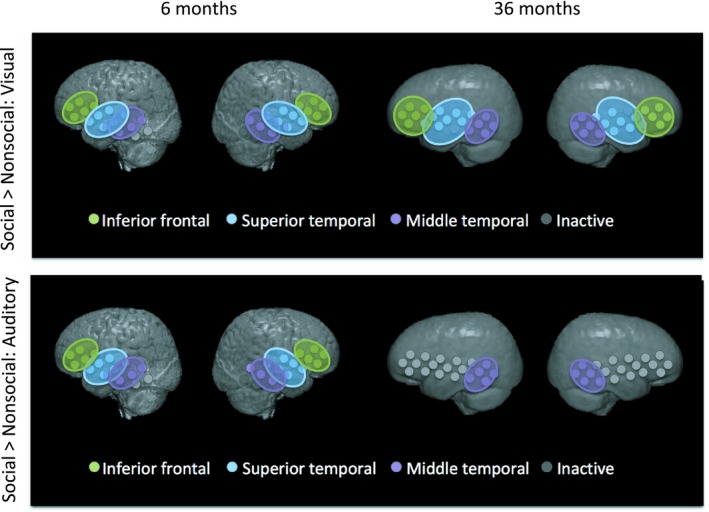
Colored disks indicate ROIs with statistically significant socially selective visual (upper panel) or auditory (lower panel) responses

Social selectivity—defined as statistically significant differences in social versus nonsocial responses in the auditory or visual domain—was seen in most ROIs at both ages (see Figure 5 for oxyHb results). Statistics for both oxyHb and deoxyHb are reported in Table 2. For the 6‐month cohort, a greater oxyHb response to social versus nonsocial stimuli in both the auditory and visual contrast was found in all ROIs. For the 36‐month cohort, all ROIs showed a statistically significant greater oxyHb response to social versus nonsocial stimuli in the visual condition, but in the auditory condition only the bilateral middle temporal ROI had greater oxyHb response to the social as compared to the nonsocial condition. DeoxyHb social contrasts were generally less strong, but still significant in the 6‐month cohort to the visual social contrast in the lIFG, rSTG and rMTG. No deoxyHb contrast was found for the auditory social stimuli in the infant cohort. In the 36‐month cohort, significant deoxyHb social contrasts to the visual condition in the bilateral STG were seen, along with a significant auditory social contrast in the rIFG. Comparing between cohorts, no significant difference between ages in oxyHb contrast magnitude was found in either the visual or the auditory condition (*ps *> 0.13).

**Table 2:** ROI social selectivity in the visual and auditory domain for each cohort

ROI	Corrected *p*‐value			*t*‐statistic (df)	
	Infant	Toddler		Infant	Toddler
*oxyHb*					
Visual					
rMTG	<0.001^c^		<0.001^c^	5.55 (73)	4.11 (101)
rSTG	0.002^b^		<0.001^c^	3.44 (71)	6.07 (102)
rIFG	0.006^b^		<0.001^c^	2.83 (73)	4.26 (103)
lMTG	<0.001^c^		0.005^b^	4.39 (72)	2.84 (100)
lSTG	0.002^b^		<0.001^c^	3.33 (71)	4.84 (103)
lIFG	0.003^b^		<0.001^c^	3.14 (72)	3.90 (103)
Auditory					
rMTG	0.015^a^		0.014^a^	2.49 (73)	2.91 (101)
rSTG	0.007^b^		0.15	2.86 (71)	1.45 (102)
rIFG	0.004^b^		0.051	3.23 (73)	2.26 (103)
lMTG	0.007^b^		0.012^a^	2.91 (72)	3.16 (100)
lSTG	0.002^b^		0.065	3.80 (71)	2.05 (103)
lIFG	0.003^b^		0.15	3.44 (72)	1.52 (103)
*deoxyHb*					
Visual					
rMTG	<0.001^c^		0.38	−4.19 (73)	−0.88 (101)
rSTG	<0.001^b^		<0.001^c^	−3.89 (71)	−4.47 (102)
rIFG	0.10		0.27	−1.71 (73)	−1.50 (103)
lMTG	0.10		0.38	−1.67 (72)	−0.98 (100)
lSTG	0.088		<0.001^c^	−1.92 (71)	−3.81 (103)
lIFG	0.016^a^		0.32	−2.73 (72)	−1.26 (103)
Auditory					
rMTG	0.90		0.17	0.12 (73)	−1.60 (101)
rSTG	0.14		0.50	−1.71 (71)	−0.68 (102)
rIFG	0.14		0.023^a^	−1.73 (73)	−2.95 (103)
lMTG	0.14		0.17	−1.81 (72)	−1.63 (100)
lSTG	0.37		0.05	−1.03 (71)	−2.43 (103)
lIFG	0.14		0.25	−2.07 (72)	−1.27 (103)

a *ps * < 0.05.

b *ps * < 0.01.

c *ps * < 0.001.

### 3.3 Relationships between neural responses and risk factors

Relationships between the magnitude of the oxyHb social contrasts and the seven risk variables of interest were calculated using bivariate nonparametric correlations to screen for entry into an omnibus regression model. Risk variables included three measures related to undernutrition: HAZ, head circumference, and elevated inflammation, two measures related to low SES: poverty and maternal education, and two measures related to the child's social environment: maternal stress, and family care. Bivariate associations between study variables are reported uncorrected for multiple comparisons, and this relatively liberal criterion ensures that all risk variables that should be included or controlled for in the omnibus test are included. Bivariate results are not interpreted but are reported to explain the construction of the omnibus regression model. All correlations are reported in Table 3.

**Table 3:** Correlations between risk factors in each cohort

		Head circum.	HAZ	Poverty index	Stress index	Family care	Maternal edu.
6 months old cohort							
HAZ	Corr.	0.126					
	Sig.	0.313					
Poverty index	Corr.	−0.201	−0.103				
	Sig.	0.096	0.408				
Stress index	Corr.	−0.035	0.310^a^	0.066			
	Sig.	0.773	0.011	0.588			
Family care	Corr.	0.072	0.035	0.014	0.065		
	Sig.	0.554	0.782	0.910	0.592		
Maternal edu.	Corr.	0.199	0.054	−0.451^c^	0.144	0.220	
	Sig.	0.099	0.663	<0.001	0.233	0.065	
CRP (log)	Corr.	0.011	−0.101	−0.191	0.056	0.053	0.217
	Sig.	0.932	0.423	0.120	0.654	0.668	0.075
36‐month‐old cohort							
HAZ	Corr.	0.308^b^					
	Sig.	0.001					
Poverty index	Corr.	−0.248^a^	−0.394^c^				
	Sig.	0.011	<0.001				
Stress index	Corr.	0.023	−0.151	0.197^a^			
	Sig.	0.820	0.127	0.046			
Family care	Corr.	0.091	0.051	−0.16	−0.053		
	Sig.	0.360	0.610	0.105	0.593		
Maternal edu.	Corr.	0.157	0.149	−0.471^c^	−0.102	0.194^a^	
	Sig.	0.111	0.131	<0.001	0.304	0.049	
CRP (cumulative)	Corr.	−0.087	−0.279^b^	−0.283^b^	0.064	−0.101	−0.201^a^
	Sig.	0.395	0.005	0.005	0.535	0.323	0.048

a *ps * < 0.05.

b *ps * < 0.01.

c *ps * < 0.001.

In the 6‐month cohort, significant correlations were seen between visual social activation magnitude in the rIFG and the poverty sum score (*r *= 0.251, *p *= 0.035), and visual social activation in the lSTG and maternal education (*r *= −0.259, *p *= 0.032). The auditory social contrast in the lSTG was significantly related to HAZ (*r *= 0.248, *p *= 0.046) and poverty (*r *= −0.284, *p *= 0.018). These significant correlations determined that poverty sum score, maternal education, and HAZ should be included in the omnibus regression test.

In the 36‐month cohort, a significant correlation was found between visual social activation magnitude in the rSTG and maternal stress sum score (*r *= 0.223, *p *= 0.024). Correlations between the auditory social contrast magnitude and risk factors were seen in the rMTG, rSTG, lMTG, lSTG, and lIFG. The auditory social contrast magnitude was related to the HAZ score (lMTG: *r *= 0.244, *p* = 0.014), poverty (rMTG: *r* = −0.341, *p* < 0.001, lIFG: *r* = −0.203, *p* = 0.038), maternal stress (lSTG: *r* = −0.237, *p* = 0.016), and family care (rMTG: *r* = −0.341, *p* < 0.001, rSTG: *r* = 0.201, *p* = 0.041). These correlations were used to determine that HAZ, poverty sum score, maternal education, maternal stress, family care should be included in the omnibus regression test. Head circumference and inflammation were uncorrelated with the social contrasts in both age groups and were therefore not included in the omnibus test.

**Figure 6:**
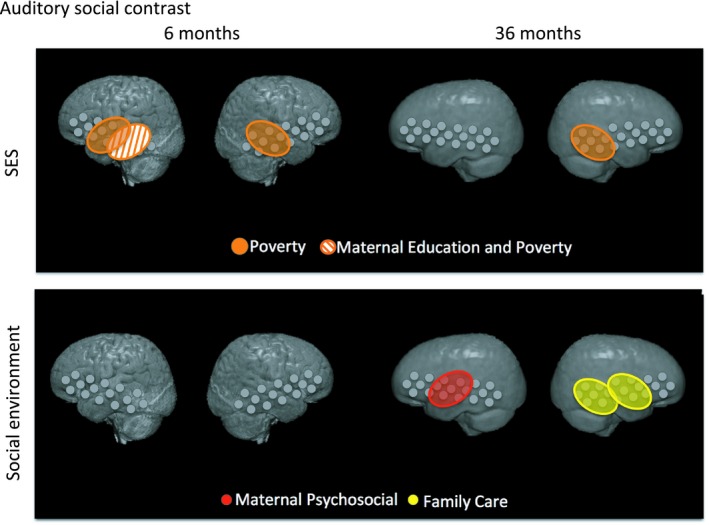
ROIs where the auditory social contrast magnitude was related to SES (poverty, maternal education) or social environment (maternal psychosocial, family care) risk scores in infants or toddlers

Risk variables that were correlated with either the visual or auditory social contrast in either group at any ROI were used to construct an omnibus regression model. This criterion eliminated head circumference and inflammation as regressors of interest, but kept HAZ, poverty, maternal education, maternal stress, and family care. The omnibus regression model was run separately for each modality, ROI, and age. Results are summarized in Figure 6. In the 6‐month group, no risk factors had statistically significant relationships with the visual social contrast magnitude after controlling for all other factors. The auditory social contrast magnitude in the rMTG was related to poverty [*b *= −0.19, *t*(60) = −2.80, *p* = 0.007]. The auditory social contrast magnitude in the lMTG was related to poverty [*b* = −0.21, *t*(59)  = −2.72, *p* = 0.008] and maternal education [*b* = −0.19, *t*(59)  = −2.95, *p* = 0.005], and the auditory social contrast in the lSTG was also related to poverty [*b* = −0.18, *t*(58)  = −2.91, *p* = 0.005]. In the 36‐month group, the visual contrast magnitude in the rSTG was related to maternal stress [*b* = 0.019, *t*(96)  = 2.00, *p* = 0.048]. This correlation between rSTG visual contrast magnitude and maternal stress was found in the bivariate analysis and remained significant when controlling for other risk factors. The auditory social contrast magnitude in the rMTG was related to poverty [*b *= −0.17, *t*(95) = −3.04, *p *= 0.003] and family care [*b* = 0.13, *t*(95) = 2.54, *p *= 0.012]. The auditory social contrast in the rSTG was also related to family care [*b* = 0.14, t(96)  = 2.25, *p* = 0.026], and the auditory social contrast in the lSTG was related to maternal stress [*b* = −0.02, *t*(97) = −2.28, *p* = 0.025]. The relationships between the auditory social contrast in the rMTG and family care or poverty were also found in the bivariate analysis, and remained significant when controlling for other risk factors. The relationship between rSTG auditory social contrast and family care was similarly seen in the bivariate analysis and remained significant. The relationship between maternal stress and lSTG auditory social contrast was found in the bivariate analysis and remained significant in the combined risk factor model.

## 4 DISCUSSION

We found greater responses to social stimuli as compared to nonsocial stimuli in both the auditory and visual domains in the 6‐month and 36‐month cohorts. These findings are consistent with prior studies using the same paradigm in similar ages, both in high‐resource (Lloyd‐Fox, Blasi, Everdell, Elwell, & Johnson, 2011; Lloyd‐Fox et al., 2018, 2009) and low‐resource settings (Lloyd‐Fox et al., 2014, 2017). Even though the data analysis used here differs from that used in prior studies, the similarities between the studies highlight the robustness of the paradigm to different analysis pipelines. The similarity of basic social selectivity responses across ages and in different cultural settings supports the paradigm as a candidate for assessing social information processing in global settings.

In the 6‐month cohort, we found that the bilateral MTG, STG, and IFG had greater activation to social versus nonsocial stimuli in both the visual and auditory domains. Similar visual social selectivity across ROIs in the IFG, MTG, and STG has been found in prior studies (Lloyd‐Fox et al., 2018). This broad activation suggests that there is differentiation of social stimuli not only in regions most linked to social processing (bilateral STG), but also in regions linked to biological motion (bilateral MTG) and general attention (bilateral IFG). We also found auditory social selectivity in all ROIs. Although auditory social selectivity has been commonly found with this paradigm (Lloyd‐Fox et al., 2011, 2018, 2014), here we see broader patterns of activation than have been found in other studies. Again we see not only responses in the social cognition hub (bilateral STG) but also in regions linked to multimodal sensory processing (MTG and IFG), auditory processing more generally (bilateral MTG), and attention (bilateral IFG). It is possible that the extended spatial extent of social selectivity is due to the greater statistical power in this study as compared to previous studies, due in turn to the larger number of participants. It is also possible that an underlying difference in this cohort as compared to prior cohorts led to broader spatial extent of the neural responses. This paradigm has been used in both high‐ and low‐resource settings with comparable results (Lloyd‐Fox et al., 2011, 2018, 2014), so it seems unlikely that the spatial extent of the response was related to the LMIC setting. However, we cannot rule out that the cohort presented here differed in some way that led to more broadly distributed social processing.

In the 36‐month cohort, we observed that the social responses differed depending on the sensory domains examined. For the visual domain, we found a broad pattern of social selectivity similar to what was seen in the 6‐month cohort, with greater activation to social versus nonsocial visual stimuli in all ROIs. While no studies have looked at 36‐month‐olds with this paradigm before, previous work does suggest that the visual social selectivity response is established between 4 and 8 months of age and extends to at least 24 months of age (Lloyd‐Fox et al., 2017). In the auditory domain, we found social selectivity in the bilateral MTG but not other ROIs, suggesting more focal social selectivity in 36‐month cohort than was found in the 6‐month cohort. As the auditory task was presented with both visual and auditory stimuli, it is possible that differences in multimodal processing with development may impact the contrast between auditory social and nonsocial conditions in the two cohorts. In young infants in high income countries, auditory processing has been shown to be dominant when auditory and visual stimuli are presented simultaneously (Robinson & Sloutsky, 2010; Sloutsky & Napolitano, 2003). Auditory dominance has been reported to extend to at least 8 years of age(Dionne‐Dostie, Paquette, Lassonde, & Gallagher, 2015; Nava & Pavani, 2013), although there is some evidence that preschoolers are able to flexibly shift their attention to auditory or visual stimuli when they are presented simultaneously (Noles & Gelman, 2012). The extent to which 36‐month‐olds are able to attend to simultaneous but incongruent audiovisual stimuli is not well understood. However, if there is improved multimodal (i.e. visual) processing in our 36‐month cohort, due to the simultaneous presentation of social visual stimuli during auditory stimuli, neither auditory condition may be perceived as truly nonsocial. Therefore, we may see reduced auditory social contrast as compared to the visual contrast as a result of the similarity between auditory conditions in the visual domain. Future studies could test for evidence of change over time by following a cohort longitudinally to see if the spatial extent of the auditory social response narrows with age. Additionally, at 36 months it may be possible to test for auditory social and nonsocial contrast without simultaneous visual presentation, which would help address the issue of multimodal integration of auditory and visual stimuli.

In both of our cohorts, we found bivariate correlations between our multiple measures of risk for poor neurodevelopmental outcomes. The strongest relationships were seen between the poverty sum score and years of maternal education in both age cohorts. These two measures are often combined into a single measure of socioeconomic status, but they suggest differential mechanisms for impacting cognitive and socioemotional development. Maternal education may more directly be related to the caregiving environment, while poverty may be more closely related to biological risk factors, therefore we wanted to keep them separate to test for differential relationships with brain function. Other relationships between risk factors differed between cohorts, with the 36‐month cohort having stronger relationships between risk factors in general. In the 36‐month cohort, the poverty sum score was correlated with every risk factor except for the family care measure, underscoring the importance of including poverty as a measure of interest when studying poverty‐related risks.

Our two cohorts, despite being recruited from the same neighborhood, also had differences in their reported levels of biological and psychosocial risk. We recruited cohorts from similar neighborhoods in an effort to ensure that the risk exposure would be similar, so that observed differences in associations between information processing and risks across cohorts likely reflect age‐related differences, rather than cohort‐specific differences in risk exposures. As shown in Table 1 most risk exposures, including the poverty index, maternal psychological stress, and maternal education are largely similar across cohorts. We note some differences, however. The 36‐month‐old children are for instance, on average, shorter for age (lower HAZ) and the caregivers of the 6‐month‐olds report engaging in fewer stimulating activities compared with the 36‐month‐old children. While these differences may reflect developmental phenomena related to growth patterns and typical age‐appropriate play activities, it is also possible that cohort (e.g. sampling) characteristics are driving the effects.

Interestingly, we note that ‘degree of poverty’, namely the cumulative index of income‐to‐needs, and housing conditions and assets, appears to show the strongest relationship with neural outcomes reflecting selectivity to social auditory stimuli as revealed by the omnibus regression test. More specifically, we find that poverty explains variation in the selective social auditory response in both 6‐ and 36‐month‐olds when controlling for other risk factors. Associations with poverty were observed in the bilateral MTG and lSTG in the 6‐month‐olds, and the rMTG in the 36‐month‐olds. Other psychosocial risks also showed associations with selective social auditory processing, but only in one of the two age groups. Maternal education, for instance, was related to selective social auditory responses in the lMTG in the 6‐month‐olds only, whereas maternal psychological stress and family care showing associations with selective social auditory responses in the 36‐month‐olds, with associations observed in the lMTG for maternal psychological stress and in the rMTG and rSTG for family care. The bilateral MTG and STG are thought to underpin social processing, perhaps explaining why we see relationships between responses in these regions and aspects of the social environment but not biological risk factors such as low HAZ or high inflammation. Two other hypotheses are that biological risk factors are impacting social processing but that the effects are too subtle to be measured with this fNIRS paradigm, or that they are yet to emerge. The fact that we only see associations between maternal psychosocial stress and family care and neural functions in the older children may suggest that 36‐month‐old children are more impacted by caregiving‐related characteristics of their psychosocial environment, or that neural impacts of the caregiving environment increase over time. At 6 months, children have been embedded in their social environment for a relatively short period of time and it may take a longer period of exposure before effects of the social environment can be detected in children's neural responses.

As for why the auditory social domain appears to be more impacted than the visual social domain in this study, more research is needed. It is known that poverty impacts language development (Pavlakis, Noble, Pavlakis, Ali, & Frank, 2015), and that responses to social non‐speech sounds in infancy are related to later brain responses to speech sounds (McDonald et al., 2019). The link, if any, between auditory social brain responses in infancy or young childhood and later language skills should be examined to determine which brain activations and risk factors would be most important to address through interventions. It may also be important to explore poverty‐related risk factors other than the ones in this study, as here we see the strongest relationships between brain and risk with poverty itself, instead of with poverty‐related risk factors that would suggest a mechanism by which poverty might impact brain function.

Limitations of the current study include the exploratory nature of the analyses linking brain function and risk measures, and the relatively limited range of poverty within and between cohorts. We suggest that follow‐up studies comparing these results in low income cohorts to higher‐income cohorts may further elucidate the impact of poverty on the developing social brain as well as tease apart relationships between correlated risk factors. A caveat should be mentioned regarding the implementation of the stimuli used. The degree of motion in the visual stimuli did differ between the social and nonsocial conditions. However, prior work using dynamic social and nonsocial stimuli has found that, at least in infants, the social‐nonsocial contrast does not appear to be driven by the level of movement in the stimuli (Braukmann et al., 2018). Overall this work has provided an important start, but more work, and particularly evaluating targeted interventions on risk factors, will provide further insight into mechanisms derailing brain development while potentially helping children in LMICs reach their developmental potential.

## CONFLICT OF INTEREST

The authors declare no conflicts of interest.

## Data Availability

The data that support the findings of this study are available from Charles A. Nelson (Charles_Nelson@harvard.edu) upon reasonable request.
