# Supplementary figures and images for: Using functional near‐infrared spectroscopy to assess social information processing in poor urban Bangladeshi infants and toddlers
Source: Dev Sci. 2019 May 17;22(5):e12839. doi: 10.1111/desc.12839 (PMC6737924; doi:10.1111/desc.12839)

## 6 month ROIs:

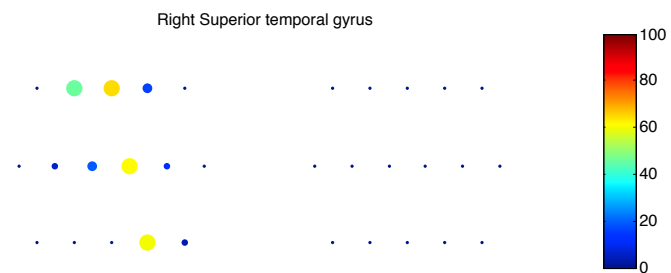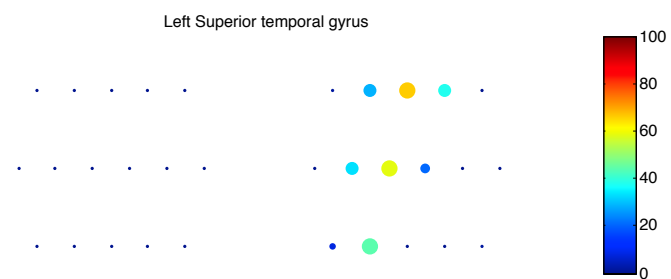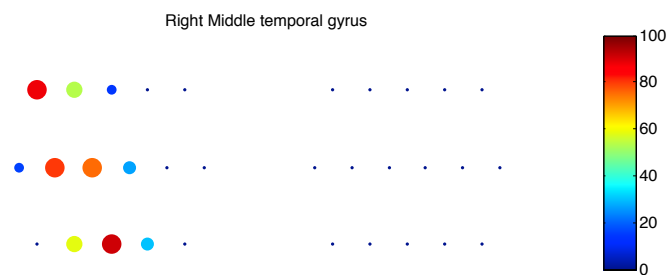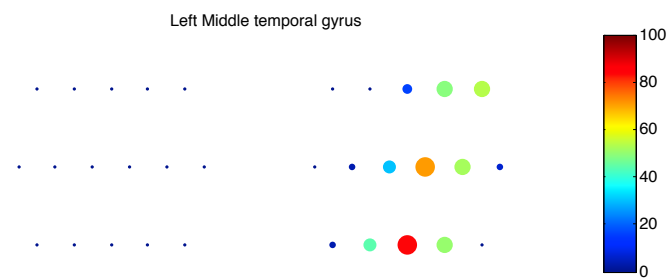

## 36 month ROIs:

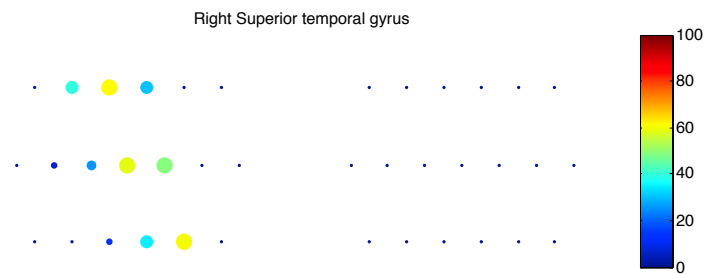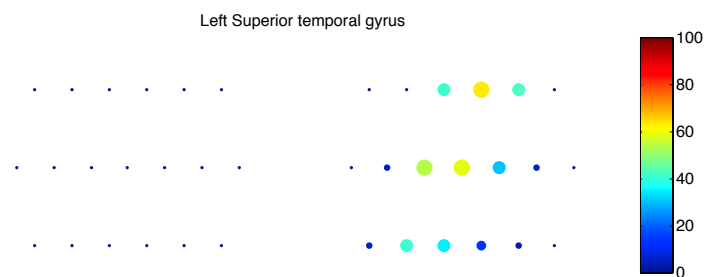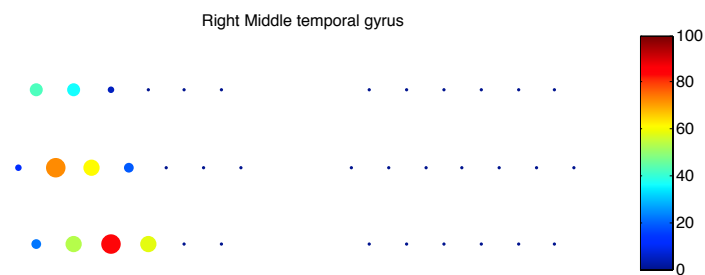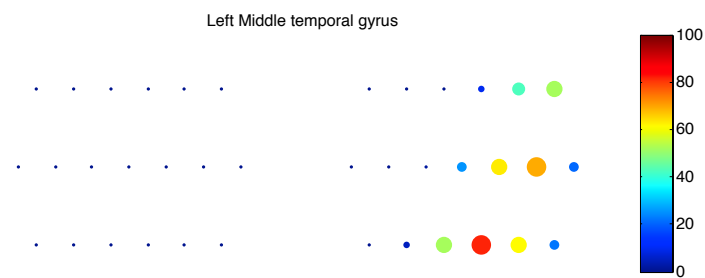

Supplement: Supplementary file 1 [file DESC-22-na-s001.pdf]
